# Do Novel Biomarkers Have Utility in the Diagnosis and Prognosis of AKI? CON

**DOI:** 10.34067/KID.0000000000000188

**Published:** 2023-06-09

**Authors:** Steven G. Coca

**Affiliations:** Barbara T. Murphy Division of Nephrology, Department of Medicine, Icahn School of Medicine at Mount Sinai, New York, New York

**Keywords:** AKI and ICU nephrology, acute tubular necrosis, kidney function, prognosis

AKI is most commonly recognized and defined by an acute increase in serum creatinine (SCr). However, SCr is an imperfect marker of AKI for a number of reasons. First, SCr cannot be a true representative of underlying GFR because of confounding factors including advanced age, increased or decreased muscle mass, excessive protein intake, and certain medications that interfere with tubular secretion of creatinine. Second, the kinetics of SCr elevation often lag behind the time of acute stressor and the onset of tubular injury. Third, if the SCr is elevated because of kidney dysfunction, an elevation may reflect either decreased glomerular filtration with or without acute tubular injury (ATI). Finally, it is believed that clinical AKI that involves ATI eventually leads to nephron dropout and fibrosis and thus will likely result in higher risk of CKD/progressive CKD compared with AKI manifested by an equal amount of decrement in GFR because of hemodynamic perturbations without tubular injury.^1^ Thus, the inadequate sensitivity, specificity, and prognostic ability of SCr in AKI have led to the search for biomarkers to accomplish the following:

- Allow for earlier and more accurate diagnosis of AKI
- Distinguish true tubular injury from decreased glomerular filtration
- Prognosticate subsequent outcomes for patients with AKI

There have been hundreds to thousands of articles published in the past two decades on biomarkers in AKI.^2^ Do we have biomarkers that accomplish the stated goals above? Moreover, as per the question posed for this specific debate, do the biomarkers have *UTILITY*? Utility implies that the biomarkers satisfy at least one of the three abovementioned criteria and that *action* on the knowledge of the biomarker will *change and improve outcomes*.

There is no existing therapy for intrinsic AKI (ATI, AKA classic acute tubular necrosis [ATN]). Indeed, we cannot apply a pan-AKI hypothesis of treatment but rather have to examine most likely responses in various clinical settings, including sepsis-associated AKI, AKI in acute decompensated heart failure (*i.e.*, cardiorenal syndrome), AKI in advanced liver disease (*i.e.*, hepatorenal syndrome), cardiac surgery–associated AKI, chemotherapy-associated AKI, and generic multifactorial AKI that occurs in hospitalized patients in various wards. As presented in the Table 1 below, there are some theoretical advantages but also many potential disadvantages to defining or using biomarkers indicative of ATI in these settings. Most certainly, there is a paucity of data on outcomes directly or indirectly related to actions taken toward biomarkers reflective of ATI.

**Table 1:** Various clinical settings for which implementation of tubular injury biomarkers may have utility

Clinical Setting	Potential Advantages	Potential Disadvantages	Evidence (Mostly Indirect)
Sepsis	Identify those most likely to require RRT earlier	More fluids may be given for ATI, which is generally not volume-responsive and will result in total body fluid overload Earlier RRT on the basis of ATI, which may not be necessary (watchful waiting may allow for recovery and avoidance of RRT)	Fluid overload is always associated with worse outcomes in AKI in ICU^3^ No benefit of earlier RRT in severe AKI, including (in some trials) AKI defined by elevated NGAL^4^
ADHF	Distinguish those with hemodynamic SCr elevations versus ATI with goal to avoid overdiuresis	Less effective decongestion in ATI because of scaling back of diuretic regimens Holding of GDMT therapies (especially SGLT2i, MRAs, and sacubutril/valsartan) on observation of ATI	*Post hoc* analyses of ROSE-AHF^5^ and CARRESS-AHF^6^ demonstrated that those with ATI by urinary biomarkers in ADHF have evidence of better decongestion and associated with lower mortality^5^ and better eGFR^6^ at 60 d
Advanced liver disease	Better up-front management (volume for prerenal; pressors for HRS; neither for ATN)	Vasoactive agents to increase BP should not necessarily be withheld from those with ATI/ATN	Biomarker-directed therapy trials have not been conducted
Cardiac surgery	Optimize hemodynamics and medications postsurgery in those with ATI	In Prev-AKI trials,^7,8^ there was more ACEi/ARB discontinuation, more fluids given, more inotropes in the intervention arms in response to elevated Nephrocheck; all of these actions have dubious role in AKI	Randomization to multicomponent KDIGO bundle of potential kidney protective strategies for those with elevated Nephrocheck reduced severity of AKI as defined by SCr metrics but has not been shown to improve clinical outcomes^7,8^
Chemotherapy	Alter dose of chemotherapy, change chemotherapy regimen	May result in delay or less effectiveness in primary treatment of malignancy	Biomarker-directed chemotherapy trials have not been conducted
General or multifactorial	Better identification of ATI on medical wards Discontinuation of potential nephrotoxins	Knee-jerk response will likely be more fluids for ATI, which is generally not volume-responsive and will result in total body fluid overload	Alert trials for SCr-based AKI have not led to improved outcomes^9^ (and in some strata, alert to AKI increased risk for mortality)^10^

ATI, acute tubular injury; ICU, intensive care unit; NGAL, neutrophil gelatinase-associated lipocalin; ADHF, acute decompensated heart failure; SCr, serum creatinine; GDMT, goal-directed medical therapies; SGLT2i, sodium glucose cotransporter 2 inhibitors; MRAs, mineralocorticoid antagonsits; HRS, hepatorenal syndrome; ATN, acute tubular necrosis; ACEi, angiotensin converting enzyme inhibitor; ARB, angiotensin receptor blocker; KDIGO, Kidney Disease Improving Global Outcomes.

Although this is not a comprehensive list of all of the theoretical benefits and disadvantages that might ensue with employment of biomarkers of ATI in these clinical settings, it serves the point to have the reader consider the nonintended consequences of having more ubiquitous testing and evidence for ATI. Most would agree that clinicians tend to favor more fluids/more hydration as beneficial for the kidneys. However, numerous studies have demonstrated the adverse consequences of fluid overload in AKI.^3^ Furthermore, ATI/ATN is usually not a volume-responsive form of AKI.^11^ Moreover, the data that have been generated to date listed in the final column of the Table 1, although far from definitive (and acknowledging that some data are indirect evidence), are not supportive of the hypothesis that more frequent ATI detection in these clinical settings will be beneficial to patients. Indeed, until it is shown in a randomized trial that action(s) in response to ATI detection with novel biomarkers leads to better outcomes,^12^ it will remain a dubious strategy.

Before closing, there are two potential scenarios for biomarkers of ATI in clinical AKI that may have utility. The first clinical setting relates to distinguishing between ATN versus acute interstitial nephritis (AIN). The urinary biomarkers TNF-*α* and IL-9 provide robust discrimination for AIN versus other etiologies of AKI.^13–15^ AIN has a specific therapy (holding of responsible agent and glucocorticoids), and thus, better identification and treatment with these novel biomarkers has the potential for utility in the cases where AIN cannot be ruled out easily (without kidney biopsy).

The second scenario relates to post-AKI care. There are millions of hospitalizations with concurrent AKI annually in the United States.^16^ Only a minority of patients receive prompt and adequate post-AKI follow-up visits with nephrologists.^17^ It would be impossible for nephrologists to meet the demand of consultations with all patients who survive AKI hospitalization. There may be a role for biomarkers to better define those at highest risk of post-AKI CKD/CKD progression.^18–20^ While bundles of care and specific therapies to target AKI to CKD transition need to be tested prospectively,^17^ the utility of prognostic biomarkers in this setting seems to have decent promise. The National Institutes of Health has funded and initiated the Caring for OutPatiEnts after Acute Kidney Injury consortium that will develop and test interventions that aim to reduce morbidity compared with usual care in stage 2 and 3 AKI survivors.
